# Metabolic Interplay between the Immune System and Melanoma Cells: Therapeutic Implications

**DOI:** 10.3390/biomedicines9060607

**Published:** 2021-05-26

**Authors:** Alice Indini, Francesco Grossi, Mario Mandalà, Daniela Taverna, Valentina Audrito

**Affiliations:** 1 Medical Oncology Unit, Department of Internal Medicine, Fondazione IRCCS Ca’ Granda Ospedale Maggiore Policlinico, 20122 Milan, Italy; alice.indini@gmail.com; 2 Medical Oncology Unit, Department of Medicine and Surgery, University of Insubria, ASST dei Sette Laghi, 21100 Varese, Italy; fg1965@libero.it; 3 Unit of Medical Oncology, Santa Maria Misericordia Hospital, University of Perugia, 06156 Perugia, Italy; mario.mandala@unipg.it; 4 Molecular Biotechnology Center, Department of Molecular Biotechnology and Health Sciences, University of Turin, 10126 Turin, Italy; daniela.taverna@unito.it

**Keywords:** melanoma, metabolic reprogramming, immunometabolism, soluble factors, tumor microenvironment, targeted therapy, immunotherapy

## Abstract

Malignant melanoma represents the most fatal skin cancer due to its aggressive biological behavior and high metastatic potential. Treatment strategies for advanced disease have dramatically changed over the last years due to the introduction of BRAF/MEK inhibitors and immunotherapy. However, many patients either display primary (i.e., innate) or eventually develop secondary (i.e., acquired) resistance to systemic treatments. Treatment resistance depends on multiple mechanisms driven by a set of rewiring processes, which involve cancer metabolism, epigenetic, gene expression, and interactions within the tumor microenvironment. Prognostic and predictive biomarkers are needed to guide patients’ selection and treatment decisions. Indeed, there are no recognized clinical or biological characteristics that identify which patients will benefit more from available treatments, but several biomarkers have been studied with promising preliminary results. In this review, we will summarize novel tumor metabolic pathways and tumor-host metabolic crosstalk mechanisms leading to melanoma progression and drug resistance, with an overview on their translational potential as novel therapeutic targets.

## 1. Introduction

Cutaneous melanoma represents 5.6% of all new cancer cases in the United States (US) [1]. According to the Surveillance, Epidemiology, and End Results (SEER) database, the 5-year relative survival rate for patients with cutaneous melanoma (all stages) exceeds 93%, ranging from 99% for localized and early-stage disease to less than 30% for metastatic disease [1]. Over the last years, the prognosis of patients with advanced/metastatic melanoma has significantly improved with the introduction of novel therapeutic strategies. These include drugs targeting the mitogen activated protein-kinase (MAPK) pathway, namely, BRAF and MEK inhibitors, in patients with BRAF mutant melanoma (i.e., approximately 50% of patients with cutaneous melanoma) [2,3]. To date, three different targeted therapies against BRAF and MEK have been approved for the treatment of advanced unresectable/metastatic melanoma, namely, vemurafenib plus cobimetinib, dabrafenib plus trametinib, and encorafenib plus binimetinib [2,3,4]. The combination of dabrafenib and trametinib has recently demonstrated to provide sustained relapse-free survival improvement as an adjuvant treatment for resected stage III melanoma, thus, being approved in this therapeutic setting [5].

Another therapeutic strategy consists of the use of monoclonal antibodies targeting immune checkpoint molecules, such as the anti-cytotoxic T-lymphocyte antigen 4 (CTLA-4) antibody, ipilimumab, and the anti-programmed cell death 1 (PD-1) antibodies, nivolumab and pembrolizumab [6,7,8,9]. Given the survival advantages with a more favorable toxicity profile, anti-PD1 treatment has become the standard first line immunotherapy strategy, while anti-CTLA-4 is commonly used in further lines of treatment in case of disease progression on previous anti-PD1 therapy. The combination of ipilimumab plus nivolumab has demonstrated to provide further improvements in survival outcomes, also in challenging subpopulations of patients (e.g., patients with brain metastases), however, with a higher rate of immune-related adverse events [8]. Anti-PD1 treatment has recently become standard of care in the adjuvant setting for patients with resected stage III (pembrolizumab or nivolumab) or stage IV melanoma with no evidence of disease (nivolumab) [10,11].

**Figure 1:**
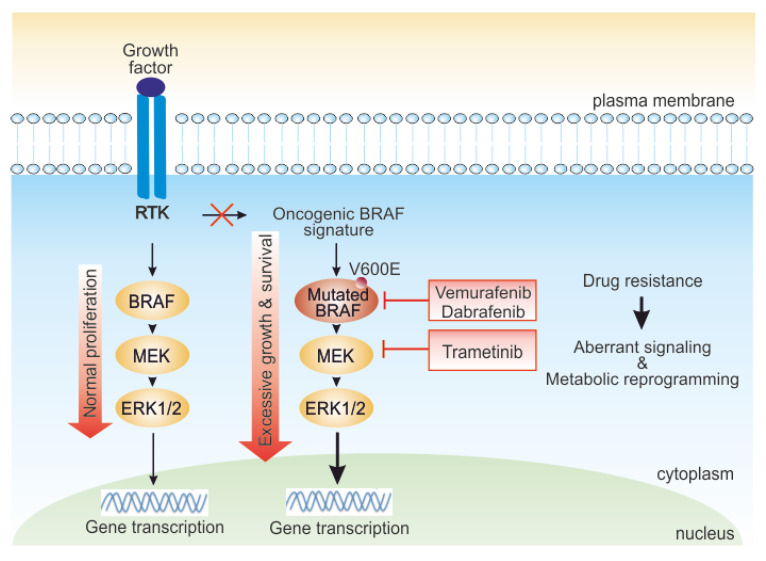
Role of the mitogen activated protein kinase (MAPK) pathway in melanoma cells and targets of BRAF and MEK inhibitors. In normal cells, external growth stimuli trigger receptor tyrosine kinase (RTK), activating the MAPK pathway kinase cascade. In BRAF-driven melanoma, mutant BRAF (*BRAF* V600E) can start signaling independently of growth factor signal to hyperactivate cellular growth. BRAF mutated melanoma responds to BRAF/MEK inhibitors-targeted therapy. However, various intrinsic or adaptive resistance mechanisms attenuate response to targeted BRAF inactivation, deregulating signaling and rewiring cell metabolism.

Despite the undoubted therapeutic advances, the majority of patients with metastatic melanoma will still die from their disease, either because of primary (i.e., innate) or secondary (i.e., acquired/adaptive) resistance mechanisms (Figure 1) [12,13,14]. Several different predictive and prognostic biomarkers have been investigated to improve patients’ selection and risk stratification, however, with disappointing and inconsistent results [15,16,17,18]. Intense research is, therefore, required to understand the biologic and molecular features of aggressive melanoma and the mechanisms underlying treatment resistance [13] to increase therapeutic possibilities.

Metabolic rewiring (i.e., glycolysis and/or oxidative phosphorylation OXPHOS pathways) in melanoma, and the metabolic crosstalk between melanoma cells and the components of the tumor microenvironment (TME), represent an intriguing field of research due to the role of metabolic process in supporting tumor survival and progression but also drug resistance [19,20,21,22,23] (Figure 1).

In this review, we explore the symbiotic relationship between melanoma and immune/stromal cells, focusing on metabolic plasticity of melanoma and immune cells within the TME and metabolic soluble factors that promote melanoma aggressiveness and immune evasion. We also discuss the ongoing clinical trials and future potential combination strategies exploiting metabolic players in cutaneous melanoma to further improve therapeutic outcomes.

## 2. Tumor Metabolic Plasticity Driving Melanoma Progression and Resistance

**Figure 2:**
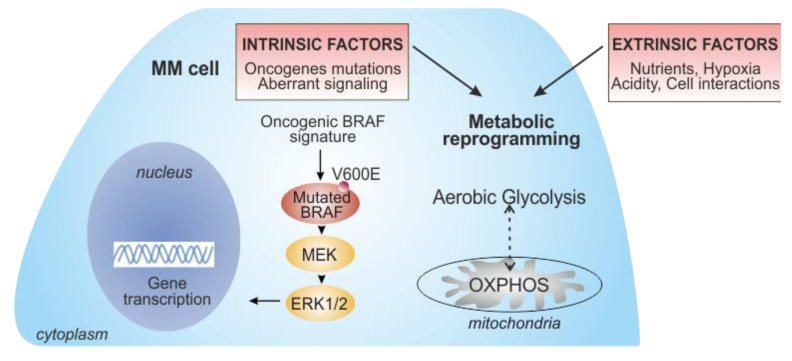
Key intrinsic and extrinsic factors contributing to metabolic reprogramming in metastatic melanoma (MM) cells. The oncogenic BRAF mutated molecular pathway, leading to the overactivation of MAPK, drives metabolic reprogramming in melanoma cells, promoting glucose metabolism (intrinsic factors). However, some melanomas rely on oxidative phosphorylation (OXPHOS), suggesting a metabolic plasticity that supports melanoma progression and resistance to drugs. During the onset of BRAF resistance, prolonged inhibition of BRAF/MEK decreases glycolysis, leading to a dependence on mitochondrial metabolism. Metabolic rewiring of MM cells is also regulated by several extrinsic factors, such as the availability of nutrients, hypoxic conditions, and acidity of the TME, as well as the interplay with stromal/immune cells within the TME. The progressive metabolic reprogramming in melanoma is accompanied by a drastic increase in tumor aggressiveness.

Metabolic reprogramming has been widely accepted as one of the hallmarks of cancer [19,24]. Tumor cells must adapt their metabolic needs to support sustained proliferation, growth, and metastatic potential [25]. To achieve this, cancer cells usually switch from a mitochondrial oxidative metabolism to a glycolysis-based metabolism, a process known as the “Warburg effect”. In 1920, Otto Warburg first defined this metabolic feature of tumors, which showed high rates of glucose uptake and lactate secretion, even in the presence of oxygen (i.e., aerobic glycolysis) [23,26,27]. This paradigm has slightly changed since it is now recognized that several tumors use OXPHOS, rather than glycolysis, as the preferential method for energy production [28,29]. Several intrinsic factors in tumor cells, mainly driven by oncogenes activation and tumor suppressor genes inactivation, together with the hypoxic condition and nutrients competition within the TME, drive the acquisition of such metabolic pathways (Figure 2) [24,30]. The BRAF oncogene has emerged as a critical regulator of these processes in melanoma cells, underlying the importance of metabolic rewiring in the pathogenesis and treatment of metastatic melanoma. In the early phase of its development, melanoma is characterized by a glycolytic metabolism [31,32,33]. BRAF mutations lead to MAPK pathway hyperactivation and subsequent stimulation of transcription factors such as MYC and hypoxia inducible factor-1α (HIF-1α), which are key regulators of glycolysis, inducing transcription of several genes involved in glucose metabolism (i.e., glucose transporter 1 (GLUT1), hexokinase (HK2), and lactate dehydrogenase (LDH)) [31,34]. In parallel, BRAF mutations actively inhibit OXPHOS, repressing expression of microphthalmia-associated transcription factor (MITF) and its target peroxisome proliferator-activated receptor gamma coactivator-1 alpha (PGC-1α), a key regulator of mitochondrial functions [35,36,37]. Treatment with BRAF and MEK inhibitors rapidly and significantly reduces the addiction of melanoma cells to glycolytic processes [31,38]. However, increasing evidence has demonstrated that melanoma is a highly heterogeneous tumor, and subsets of melanoma possess an oxidative metabolism, correlating with poorer survival, progression, and metastasis [21,22,37,39]. Higher OXPHOS is driven by elevated expression of PGC-1α. These tumors show an improved tolerance to the detrimental effects of reactive oxygen species (ROS), indicating their increased ability to survive under conditions of oxidative stress [39]. It is, therefore, clear that both glycolysis and OXPHOS play a significant role in metabolic reprogramming of melanoma cells, and that there is a dynamic switch and plasticity between these two metabolic phenotypes [21,28,40]. In addition, some melanomas show a “hybrid” glycolysis/OXPHOS metabolic phenotype, meaning that tumor cells have the flexibility to use different energy sources and nutrients to adapt their growth according to different TME conditions [33,39,41].

Metabolic plasticity is essential during the onset of resistance to BRAF inhibitors. In resistant patients there is a switch from glycolysis to a mitochondrial metabolism [34,42,43]. One hypothesis to explain this phenomenon is that this metabolic oxidative profile pre-exists in some cell clones, and that treatment leads to the selection of these cell clones. Another possibility is that tumor cells acquire resistance as a consequence of treatment, following drug exposure. The main molecular player of this metabolic phenotype is the reactivation of the MITF/PCG-1α axis, driving increased mitochondrial content, mitochondrial activity, and mitochondrial oxidative capacity [37,39]. This adaptive metabolic program limits the efficacy of BRAF inhibitors and is responsible for adaptive resistance [37,39,43,44,45]. Overall, it is clear that metabolic plasticity confers to melanoma cells a significant advantage to adapt their growth to different environmental conditions and to increase their survival, even under drug-imposed selective pressures.

## 3. Metabolic Exchanges within the TME: Soluble Factors

Metabolic conditions in the TME are influenced by many factors, including oxygen levels, gradients of nutrients, soluble molecules, tissue vascularization, interactions between tumor and stromal/immune cells, and systemic metabolism (Figure 2). Recent detailed reviews summarized this complex network of metabolic crosstalk within the TME [46,47,48,49,50]. Here, we discuss the main players of this complex network, focusing on melanoma–TME metabolic crosstalk.

### 3.1. Lactate Shuttling in Cancer Cell Metabolism

The concept describing lactate as solely a hypoxic waste product has changed in the last decades. It is clear that lactate is both a potent fuel and a critical signaling molecule, and it is constantly being produced and circulated throughout the body, even in the presence of adequate O_2_ levels [51]. For these reasons, it is widely accepted that lactate is a key intermediate metabolite in cellular metabolism. The “lactate shuttle hypothesis”, originally introduced by George Brooks in 1986 [52], describes how in normal physiology, as well as in pathophysiology, lactate shuttling between and among cells satisfies at least three purposes for lactate: (i) a major energy source, (ii) the major gluconeogenic precursor in Cori cycle, and (iii) a signaling molecule with autocrine, paracrine, and endocrine-like properties [51]. Exchanges between lactate “producer” and “consumer” exist within cell “intracellular lactate shuttle” and among cells, tissues, and organs “cell–cell lactate shuttle” [53]. Lactate can be exported or up-taken across biological membranes through monocarboxylate transporters (MCTs). Importantly, MCTs are bidirectional, allowing for tissues to switch between lactate release and uptake depending on changes in concentration and pH. Usually, MCT1 is typically expressed in cells importing lactate, while MCT4 is expressed in cells exporting lactate [54].

Tumor mass, as previously discussed, is characterized by a metabolic plasticity and heterogeneity among tumor cells; moreover, different tumor types rely on multiple metabolic pathways. Lactate is an essential metabolite present in the TME and can be shuttled to and from cancer cells and is highly correlated with cancer aggressiveness and poor survival [55,56,57]. The traditional “Warburg Effect” describes glycolytic tumors relying highly on glucose uptake (via GLUTs transporters) with subsequent lactate exportation (via MCT4) in normoxia. In this context, other cancer cells within the tumor mass and/or tumor stromal cells can then take up lactate (via MCT1) to fuel their oxidative metabolism and activate signaling [57,58]. Increasing knowledge on cancer metabolism supports the idea that lactate production, “lactagenesis”, is the purpose of the Warburg Effect. As discussed by San-Millan and Brooks in 2017, lactagenesis is a highly orchestrated effort from oncogenes and tumor suppressor mutations for continuous glucose utilization to produce lactate, involving five major steps: (i) increased glucose uptake through upregulation of GLUT transporter expression; (ii) upregulation of glycolytic enzyme expression; (iii) decreased mitochondrial respiration; (iv) increased lactate production, accumulation, and release; and (v) upregulation of MCT expression for further lactate shuttling and promotion of carcinogenesis [57]. Secreted lactate is necessary for supporting angiogenesis, immune escape, cell migration, metastasis, and metabolic self-sufficiency of cancer cells [57,59]. On the contrary, in the “Reverse Warburg Effect” theory, lactate is produced by glycolytic stromal cells and utilized by cancer cells, via the tricarboxylic acid (TCA) cycle and OXPHOS, as a major source of energy. Overall, lactate plays a critical role in several aspects of tumor biology, including in melanoma, as reviewed in the next sections.

### 3.2. The Role of Lactate: TME Acidification

The acidification of microenvironment is a hallmark of melanoma altering metabolic adaptation, proliferation, survival, migration, and invasion [44]. The upregulation of glycolysis in melanoma leads to protons and LDH-dependent lactate generation [60]. Protons, as lactate, are transported out of cancer cells through MCT4 [54,60]. Hypoxia, resulting also both both tumor and endothelial cells’ high oxygen consumption, sustains acidosis through the upregulation of the glycolytic pathway, mostly linked to the stabilization of HIF-1α [60,61]. Over-expression of GLUT1 and MCT4 are significantly correlated with progression from primary tumor to lymph node metastasis in a cohort of patient-derived melanoma samples, suggesting that the Warburg phenotype, lactate and protons secretion, drastically alters the melanoma microenvironment, facilitating angiogenesis, promoting melanoma metastasis, and suppressing the immune system [62,63].

**Figure 3:**
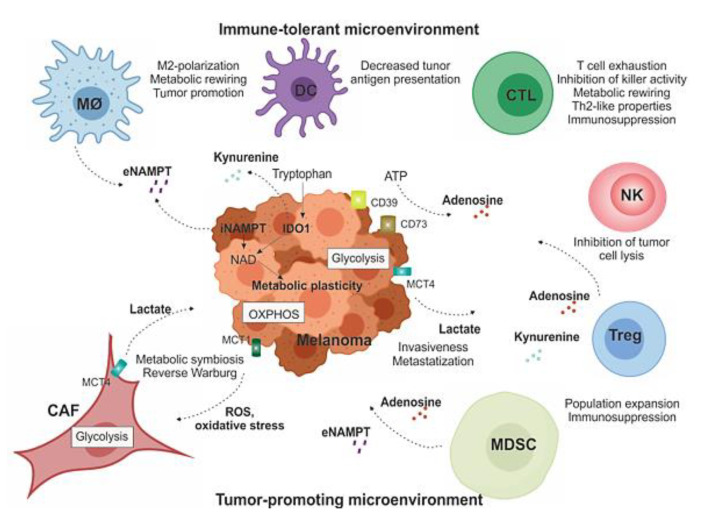
Soluble factors and immunometabolic interplay within the TME. Melanoma is usually surrounded by a wide array of stromal cells (CAFs) and infiltrating immune cells of both innate and acquired immunity, such as MDSCs, MO-TAMs, DCs, NK cells, and T lymphocytes. They form complex interactions and exchange of soluble factors with melanoma cells that modulate metabolic plasticity of cellular components and support tumor growth by creating a tolerogenic environment that enables cancers to evade immune surveillance and destruction, as detailed in the text. MO-TAM: tumor-associated macrophages; DC: dentric cell; CTL: cytotoxic T lymphocytes; NK: natural killer cell; Treg: T-regulatory lymphocyte; MDSC: myeloid-derived suppressive cells; CAF: cancer-associated fibroblasts.

In order to promote melanoma invasiveness and metastasization, lactate can contribute to tumor escape from immune responses by altering cytotoxic T lymphocytes (CTLs) metabolism and function [64,65], as summarized in Figure 3. High levels of lactate are associated with a significant decrease of CD8^+^ T and natural killer (NK) cell number and activity, both in vitro and in vivo [66]. Furthermore, lactate can also induce macrophage phenotype plasticity, promoting pro-tumoral M2-like features [62,67]. In addition, it prevents the maturation of dendritic cells (DCs), resulting in increased immunosuppressive IL-10 cytokine levels in the TME [62].

Melanoma microenvironment acidification hampers immunotherapy response [44]. LDH serum level is a well-known prognostic factor in melanoma, and it also affects response, progression-free survival (PFS), and overall survival (OS) of melanoma patients treated with immune-checkpoint inhibitors [68]. Therefore, the efficacy of immunotherapy could be improved by counteracting microenvironment acidification and lactate extracellular accumulation, as suggested by recent studies [68,69,70].

### 3.3. The Reverse Warburg Effect: Melanoma Cells and Fibroblasts Crosstalk

Metabolic reprogramming involves not only cancer cells but also cellular components of the TME, namely, cancer-associated fibroblasts (CAFs), the most abundant pro-tumoral population within tumor stroma [41,46,71]. The progressive acidification of TME and hypoxic conditions modify the metabolic interactions between cancer cells and stroma. CAFs are reprogrammed toward a glycolytic phenotype upon interaction with cancer cells, an effect called “Reverse Warburg”, Figure 3 [72,73,74]. ROS produced by cancer cells further stimulate CAFs glucose upload and lactate secretion via MCT4 [75,76]. In turn, lactate and metabolites secreted by CAFs can be taken up by tumor cells via MCT1 to feed into the TCA cycle for OXPHOS-mediated energy production [77]. This Reverse Warburg effect was initially reported in a variety of cancers, including prostate and breast [46,74], and has been confirmed also in melanoma [21,39,78]. Increasing evidence has highlighted the contribution of CAFs to disease progression, metastasis, and drug resistance in melanoma, via both direct cell–cell interaction and chemical interplay (CAF’s secretome) to distant cells [79,80,81]. CAFs-tumor crosstalk is mediated by secretion of extracellular vesicles (EVs), such as exosomes, important factors in pre-metastatic niche formation [82]. The composition of exosomes is complex and not fully elucidated; however, it includes proteins, lipids, metabolites, nucleic acids, and microRNA, which can be trafficked in circulation and internalized by recipient cells to exert their effect [46,83]. Understanding the metabolic interplay between CAFs and other stromal cells with melanoma can determine drivers of cancer progression and potentially lead to the discovery of prognostic and predictive cancer biomarkers and novel anti-cancer therapies. Moreover, these findings highlight that melanoma cells can shift from glycolysis to OXPHOS and vice versa, depending on the TME conditions and interaction with microenvironmental populations, and prompt further research to develop more effective glycolysis and/or OXPHOS inhibitors that can be associated with conventional therapies.

## 4. Immunometabolic Interplay within the TME

Immune cells, together with stromal cells, are critical components of the TME. There is growing interest in studying the dynamic metabolic interactions occurring between melanoma cells and the immune cells, as summarized in Figure 3, to discover potential novel targets to combine with immunotherapy. Additionally, immune cells undergo different metabolic reprogramming mechanisms and adapt their metabolic pathways to external conditions and upon interaction with cancer cells [49,84,85]. For example, M1 anti-inflammatory macrophages exhibit a glycolytic phenotype, while tumor associated macrophages (TAMs) or M2-like macrophages cells utilize OXPHOS as their main source of adenosine triphosphate (ATP), and neutrophils prevalently use glycolysis [84,85]; on the contrary, DCs extensively rely on OXPHOS to produce ATP, to switch toward a glucose metabolism upon activation [62]. T cells possess metabolic plasticity depending on their activation state and the subclass of T cells to which they belong: activated effector T cells adopt a glycolytic phenotype [77], T regulatory cells (Tregs) are highly oxidative cells, while regulatory T helper-17 (Th17) cells depend on glycolysis [84,85,86].

The crosstalk between these immune cell populations and cancer cells can impact and deregulate metabolic pathways affecting immune responses and creating an immunosuppressive TME. In addition, the availability of nutrients within the TME and the presence of soluble metabolites and enzymes secreted by both tumor and immune cells in the extracellular environment can alter the phenotype and the functionality of immune cells.

### 4.1. Nutrient Availability and Metabolic Competition between Tumor and Immune Cells

The nutrient competition among cells within the TME can influence tumor cell growth, survival, and aggressive features. At the same time, the abundance or the deprivation of glucose, lactate, glutamine, amino acids, fatty acids, and other metabolites, as well as growth factors, significantly affect immune cell functions, leading to cancer progression [87]. The most evident and studied effect of nutrient competition within the TME is on T cell functions [88]. Cancer cells can evade the immune system by triggering T cell dysfunction, a condition called exhaustion, or by activating immune checkpoints that inhibit T cell function. Tumors can dampen T cell function by competing for glucose [89,90]. Different papers have revealed that aerobic glycolysis in tumors results in a glucose-poor microenvironment causing T cell exhaustion, demonstrating that metabolic competition, as a distinct mechanism, can lead to T cell hyporesponsiveness. In 2015, Chang et al. demonstrated that glucose consumption by glycolytic tumors can metabolically restrict T cells, which completely depend on aerobic glycolysis to exert effector activities [91,92], directly dampening their effector function and allowing tumor progression [93]. Moreover, tumor-derived lactate can also suppress T cell function by blocking lactate export [64], which disrupts their ability to maintain aerobic glycolysis. As a mechanism of adaptation in a glucose-poor microenvironment, T cells preferentially differentiate into Treg lymphocytes, probably because their oxidative phenotype is metabolically suited to survive in this environment [94].

It has been described that the competition for glucose can affect tumor infiltrating lymphocytes (TILs) activity in melanoma. Glucose deprivation increases secretion of transforming growth factor-β (TGF-β), decreasing concentration of interferon-γ (IFN-γ), which is essential for cytotoxic activity of T cells and to inhibit growth of murine B16 melanomas [95,96]. Interestingly, IFN-γ translation can be inhibited by the glycolytic enzyme glyceraldehyde 3-phosphate dehydrogenase (GAPDH) when it is not engaged in glycolysis, highlighting the importance of glucose metabolism for maintenance of T cell function [93]. Recently, a connection between the expression of PD-L1 and PD-1 and glucose metabolism has been evidenced in melanoma and in other solid tumors. Immune-checkpoint molecule expression not only suppresses T cell function but also enhances aerobic glycolysis in cancer cells, further limiting the availability of glucose for T cells, increasing their dysfunctions [97]. Increasing data suggest that checkpoint blockade antibodies, affecting glucose metabolism, might be more effective against tumors with higher glycolytic flux [88,92]. For these reasons, the glycolytic rate of a tumor could be used as a prognostic/predictive tool to determine the efficacy of these treatments. Therefore, glucose metabolism through the glycolytic pathway is central in shaping T cell responses and is emerging as an ideal target to improve the efficacy of cancer immunotherapy [92]. In addition, it has been shown that enhancing fatty acid (FA) catabolism in conditions of low O_2_ and glucose improves TILs’ ability to kill cancer cells and that the use of peroxisome proliferator-activated receptor (PPAR)-alpha agonist enhances the therapeutic effect of PD-1 blockade in melanoma [98].

Similar to glucose metabolism, amino acid metabolism can play a regulatory role in T cell activation. For example, tumor indoleamine 2,3-dioxygenase (IDO), an enzyme that converts tryptophan to kynurenine, has been shown to deplete the essential amino acid, tryptophan, in the microenvironment, resulting in T cell inhibition [99], as detailed later in this review.

### 4.2. Adenosine Metabolism: A Critical Immunosuppressive Metabolite

A major feature of melanoma cells is their ability to escape the immune surveillance. Adenosine (ADO) is one of the main metabolites present in the TME that generates immunosuppressive conditions [100,101,102]. Metabolic stress and cell damage caused by hypoxia and inflammation lead to an enhanced hydrolysis of ATP into ADO through the enzymatic activity of two cell-surface ectonucleotidases, CD39 and CD73 [103,104,105,106]. Either produced by tumor cells and/or by immune suppressive cells, ADO accumulates in tumor tissues where it suppresses T effector cell functions, reducing their proliferation, cytotoxic activity, and pro-inflammatory cytokine secretion, including IFN-γ, tumor necrosis factor (TNF)-β, and IL-2, by binding to purinergic receptors A2aR and, partially, A2bR [101,107,108,109,110,111,112]. These receptors are Gs-coupled receptors that, by increasing intracellular cyclic AMP (cAMP) levels, mediate the immune suppressive effects of ADO, facilitating tumor progression. In addition, A2aR stimulation reduces the expression of CD25 and CD40 ligand (CD40L) and increases the expression of PD-1 and CTLA-4 on T cells [113]. ADO also promotes immunosuppression by increasing IL-10 secretion and expression of immunosuppressive proteins, such as IDO, TGF-β, and arginase-2, promoting peripheral tolerance by inducing T-cell anergy and also metabolic dysregulation [112,114,115,116].

By inducing a dysfunction in the immune responses, the CD39/CD73/ADO axis is apparently relevant for melanogenesis [117,118,119,120]. ADO interferes with signals mediated by IL-2 receptor and exerts a direct anti-proliferative effect on naive CD4^+^T and CD8^+^T cells in melanoma TME [108]. Therefore, ADO has been suggested as a key player for melanoma cells escaping from adaptive immune control (Figure 3) [121]. Immune and melanoma cells express both CD73 and ADO receptors. A2aR expression in human melanoma cell lines was originally reported by Merighi et al. This study demonstrated that ADO enhances melanoma cell proliferation through A2aR activation [122]. The first evidence of the role of A2aR in vivo in the control of melanoma growth has been reported by Ohta et al., showing that 60% of A2aR deficient mice completely rejected established immunogenic tumors by anti-tumor CD8^+^T cells [107]. These results were confirmed some years later by Waickman et al. using a different disease model (i.e., lymphoma), suggesting that A2aR is an attractive target for tumor immunotherapy that synergizes with other immunomodulatory approaches [123,124].

Recent findings have demonstrated that the activation of the MAPK pathway leads to CD73 over-expression on the surface of melanoma cells, thus, promoting an invasive phenotype. Conversely, CD73 reduction is followed by blocking the BRAF/MEK signaling [125,126,127]. Moreover, recent studies have also demonstrated that Treg and myeloid-derived suppressor cells (MDSCs) lead to the up-regulation of CD73/CD39 expression in both primary melanoma and lymph nodes, therefore, enhancing an immunosuppressive function [128,129]. In addition, high basal levels of soluble CD73 have been associated with low response rates in melanoma patients receiving immunotherapy, suggesting that CD73 potentially represents a prognostic biomarker of survival during treatment [130].

Overall, these findings provide the rationale for future therapeutic strategies mostly aimed at inhibiting CD73 signaling and A2aR blocking, in combination with targeted therapies and/or immunotherapy [102,112,124].

### 4.3. Indoleamine-2,3-dioxygenase (IDO)-Kynurenine Metabolism

Another critical player in driving immune tolerance is IDO enzyme [131,132,133,134]. Together with extrinsic suppression of CD8^+^ T effector cells by Tregs and engagement of the inhibitory receptor PD-1 by the ligand PD-L1, the deregulation of IDO represents a key mechanism promoting immunosuppression in melanoma (Figure 3) [135,136]. IDO is a cytosolic, heme-dependent enzyme responsible for the rate-limiting step of de novo nicotinamide adenine dinucleotide (NAD) synthesis from tryptophan in extrahepatic tissues. By catalyzing the initial and rate-limiting step of tryptophan degradation, IDO reduces the local tryptophan concentration and produces immune modulatory tryptophan metabolites [137,138]. In particular, cells expressing IDO produce the tryptophan catabolite kynurenine, which regulates immune functions by interacting with the aryl hydrocarbon receptor (AhR) expressed by T cells, Tregs, and DCs [139]. Kynurenine was recently shown to favor immunosuppression in the TME, leading to induction of T-cell anergy, apoptosis, increased conversion of naïve CD4^+^ T cells into Tregs, and polarization of DCs and macrophages toward an immunosuppressive phenotype [131,134,140,141,142,143]. Expression and activity of IDO on tumor and immune cells can be modulated by several signaling systems, including the engagement of toll-like receptors (TLRs), tumor necrosis factor superfamily members (TNFRs), interferon beta receptor (IFNBR), interferon gamma receptor (IFNGR), and transforming growth factor beta receptors (TGFBRs), which are able to induce or maintain IDO expression. NF-KB activation is a central downstream signal of these pathways regulating IDO expression [144].

Increased expression of IDO was associated with poor survival outcomes in patients with ovarian, lung, colorectal, and breast cancer; brain tumors; and melanoma [140,145,146,147,148]. Melanoma cells can express IDO and directly mediate T cell and NK cell cytotoxicity [149]. Spranger et al. demonstrated that IDO expression, together with PD-L1, is mediated by signals, such as IFN-γ, derived from TILs within the TME [135]. In melanoma patients and in mouse models, IDO is expressed by antigen processing cells (APCs) in tumor draining lymph nodes [150,151]. In a cohort of patients with early stage (i.e., stage I–II) melanoma, IDO expression in the sentinel lymph node was an independent negative prognostic factor for PFS and OS [152], as well as its expression in primary melanoma [148]. Moreover, a positive correlation between the high expression of IDO and clinical response to anti-CTLA-4 therapy in melanoma has been reported [153].

According to its role in driving immunosuppression, IDO has become a valid target in cancer therapy over the last years [154,155]. Competitive inhibitors of IDO are currently being tested in clinical trials in patients with solid cancer, including melanoma, with the aim of enhancing the efficacy of conventional chemotherapy, vaccines, or checkpoint inhibitors [156]. Several IDO1 inhibitors have been developed and are currently under clinical development [157,158]. Moreno et al. demonstrated that targeting IDO with the competitive inhibitor 1-methyl-tryptophan (1-MT) retards the proliferation of melanoma cells in vitro [159,160]. Although monotherapy with 1-MT has little effect on the growth of subcutaneous melanoma B16-F10 tumors, 1-MT sensitizes the tumors to chemotherapy and whole-body radiation [161]. While promising, additional exploration is required to further define how IDO mediates immunosuppression in melanoma and whether or not 1-MT can be combined with currently approved therapies. To date, the most advanced IDO inhibitor is epacadostat, a highly specific IDO1 inhibitor, which has already been tested in several clinical trials [162,163]. However, data of epacadostat in combination with immunotherapy in patients with melanoma have failed to demonstrate a significant survival benefit (see further) [164]. A better understanding of IDO biology could lead to contrast the compensatory mechanisms unleashed in the tumor cell by blocking IDO. Clinical trials with a translational background can help improve the dynamic changes of tumor metabolic mechanisms during treatment. However, IDO1 pathway remains a relevant target to block in order to improve the efficacy of cancer immunotherapy.

### 4.4. Metabolic Enzymes/Cytokines: The Role of Nicotinamide Phosphoribosyltransferase (NAMPT)

NAD is an essential cofactor for redox reactions and the substrate for NAD-consuming enzymes, including sirtuins, activating genetic and epigenetic pathways [165,166]. Dysregulation of NAD levels is a contributing factor in the pathogenesis of several diseases, including cancer [167,168,169]. Given that NAD-dependent processes catabolize the molecule, permanent NAD synthesis is required in actively proliferating cells. For this reason, cancer metabolism rewiring is often accompanied by the upregulation of NAMPT, the key-limiting enzyme that catalyzes the first reversible step in NAD biosynthesis from nicotinamide (Nam) [165,168,170,171,172]. NAMPT is highly regulated at transcriptional levels; for example, the oncogene c-MYC regulates the expression of NAMPT, enhancing glycolysis and lactate production, leading to the Warburg effect [34,173,174]. Intriguingly, NAMPT has a second life outside the cell creating immune suppressive and pro-tumor conditions [175,176,177,178]. Extracellular (e)NAMPT levels are increased in many tumors, suggesting that the molecule is a novel player in tumor–host interactions. It binds TLR4, recently identified as a receptor [179,180], on tumor or immune cells and activates several signaling pathways [166,181]. For these reasons, NAMPT was the first NAD-biosynthetic enzyme (NBE) for which a clear potential as a therapeutic target in both solid and hematologic tumors was demonstrated. NAMPT-specific inhibitors reduce NAD levels by inhibiting energy metabolism pathways, such as glycolysis, TCA, and OXPHOS, contributing to the suppression of cancer cell proliferation [178,181,182]. Unfortunately, the NAMPT inhibitors (NAMPTi) FK866 and GMX-1778 both failed in clinical trials [182], likely because they were used in unselected patients, suggesting that it is very important to select tumors addicted to NAMPT activity.

In the last 10 years, increasing evidence has supported a driving role of NAMPT in melanoma progression and drug resistance [45]. NAMPT was first identified as over-expressed in melanoma lesions compared to benign lesions at the transcriptional and protein levels [183,184,185].

Audrito et al. extensively demonstrated the driver role of the NAMPT/NAD axis in the acquisition of resistance to BRAF inhibitors: (i) NAMPT appeared to be the master regulator of NAD biosynthesis in resistant melanoma cells, a key element involved in metabolic reprogramming [45,186]; (ii) melanoma patients, including those resistant to BRAF inhibitors, showed increased tissue and serum expression of NAMPT as compared to healthy controls or to patients with localized disease. Furthermore, patients with high eNAMPT levels have an overall reduced survival [186,187]; (iii) NAMPT over-expression recapitulates BRAF inhibitors resistance phenotype plasticity [188], data confirmed also by Ohanna et al. [189]; (iv) NAMPT targeting leads to NAD and ATP depletion, decreasing cell survival and reduced tumor growth in vitro and in melanoma xenografts in immunocompromised mice [186]. The over-expression of NAMPT is associated with the oncogenic activation of MAPK pathways due to BRAF mutations. Several transcription factors activated by this oncogenic signaling (including MYC, STAT3 and 5, NK-kB, and others) can bind NAMPT promoter and induce its transcription [173,186,189]. Overall, these data confirm that NAMPT plays a central role in the phenotypic plasticity of melanoma, becoming a novel therapeutic target in the clinical setting.

The role of eNAMPT in melanoma TME remains an open issue in this field. eNAMPT can act on tumor cells activating signaling pathways that support their proliferation, including MAPK, AKT, NF-kB, as shown in [190]. Paracrine effects of eNAMPT on stromal and immune cells were described in other tumor models. For example, eNAMPT is able to polarize macrophages in M2-like type in chronic lymphocytic leukemia [191,192] and forces the mobilization of immature MDSC and enhances their production of suppressive nitric oxide in fibrosarcoma and breast carcinoma mouse models [193]. A recent paper highlights NAMPT as a critical molecule in priming pro-tumor functions of tumor-associated neutrophils (TANs) in melanoma [194], including tumorigenic conversion of TANs and their pro-angiogenic switch [194]. Lastly, a recent paper first provided evidence for a direct functional correlation in liver cancer between the expression of PD-L1 and NAD-NAMPT axis, suggesting an association between NAMPT expression and an immune escape signature [195]. NAMPT could become a predictive marker of anti-PD-L1 therapy, and this would be an important finding if confirmed also in melanoma, thinking to combination therapy with NAMPT inhibitor and/or blocking antibody [175,196,197] with targeted and immunotherapy.

## 5. Therapeutic Perspectives: Targeting Melanoma Metabolism

To date, several metabolic targets have been investigated in advanced/metastatic melanoma, most of which are in combination with standard therapeutic strategies, with the aim to avoid or delay the onset of resistance mechanisms to immunotherapy and targeted therapies. However, despite some promising preclinical results, the efforts to target metabolic pathways for the treatment of melanoma are still in a preliminary phase and have not modified the available treatment strategies.

### 5.1. Immunotherapy and Metabolic Targets

There is a strong link between cancer cell metabolism and T cell functions, as previously discussed. Several metabolic alterations and adaptive mechanisms lead to T cell differentiation, proliferation, and tumor-promoting phenotypes. For these reasons, increased attention is focused on the combination of immune checkpoint inhibitors with agents targeting metabolic reprogramming [88].

CTLA-4 signaling inhibits glycolysis, preventing activation and differentiation of naive CD8^+^ T cells [198]. PD-1/PD-L1 signaling has important metabolic effects on T cells, leading to impaired mechanisms of energy generation and macromolecules synthesis, and reduced cytokine secretion through the inhibition of glycolysis and upregulation of FA oxidation [93]. PD-L1 signaling has direct metabolic effects on cancer cells: in response to anti-PD-L1 agents, glucose uptake and lactate extrusion are decreased, suggesting that altered PD-L1 expression directly impairs T-cell metabolism, along with favoring cancer cell metabolic reprogramming. As a consequence, treatment with anti-PD1/PD-L1 antibodies succeeds in restoring the metabolic balance in favor of T cells [88,92], as previously mentioned. Moreover, immune checkpoint molecule expression largely depends on the TME metabolic conditions and, specifically, on extracellular adenosine levels, tissue hypoxia, and T cells metabolic stress [199]. This evidence suggests that immunotherapy has the potential ability to differentially target cancer and T cells through shared metabolic requirements.

Focusing on the CD73-adenosine axis, two different therapeutic strategies have been investigated in melanoma, namely, small molecule inhibitors and monoclonal antibodies. Combining CD73 inhibitors with both anti-CTLA-4 and anti-PD1 antibodies significantly increased the effect of immunotherapy through an increased frequency of tumor-infiltrating CD8^+^ T cells, suppressed Treg accumulation within tumor tissues in murine melanoma models, and enhanced T cell responses [200,201]. This effect was more pronounced in tumors with high expression levels of CD73, suggesting that CD73 could be used as a potential biomarker to select patients who are more likely to benefit from combination therapy. However, these findings should still be verified and confirmed in the clinical setting.

In addition, as previously discussed, targeting IDO could also be a novel therapeutic strategy in combination with immunotherapy. The largest phase III trial to date evaluating the IDO inhibitor, epacadostat, in combination with the anti-PD1 antibody, pembrolizumab, in advanced melanoma patients, was the ECHO-301/KEYNOTE-252 [163]. This double-blind trial randomized over 700 patients with unresectable or metastatic melanoma in a 1:1 ratio to receive pembrolizumab in combination with epacadostat or placebo. Despite the promising preclinical findings and early-stage data from the phase I/II trial ECHO-202/KEYNOTE-037, the final results of the phase III trial did not show any survival benefit in patients treated with the combination therapy as compared to pembrolizumab alone [163,164]. These disappointing results suggest that some points still need to be elucidated, mainly, which is the best clinical setting for IDO1 inhibition to be used.

### 5.2. Targeted Therapy and Metabolic Targets

As discussed before, BRAF oncogene plays a key role in the metabolic reprogramming of melanoma cells. Moreover, metabolic adaptation both occurs in response to MAPK pathway inhibition and also contributes to resistance mechanisms during treatment with BRAF and MEK inhibitors [202].

The combination of BRAF and MEK inhibitors with drugs targeting OXPHOS is a promising strategy to enhance the effect of MAPK pathway inhibition and overcome drug resistance [43,203,204,205]. Several drugs increase the efficacy of MAPK inhibitors to induce cell death in melanoma by acting on mitochondrial bioenergetics of tumor cells. Nonsteroidal anti-inflammatory drugs (NSAID), diclofenac and lumiracoxib, increased the anti-glycolytic impact of BRAF inhibitors and prevented RAF-inhibitor induced metabolic reprogramming towards OXPHOS, thus, leading to an enhanced sensitivity of melanoma cells to BRAF inhibitors and delayed onset of treatment resistance [206]. Gamitrinib, an inhibitor of heat shock protein (HSP)90, represents another possible strategy to overcome drug resistance to MAPK inhibitors [203] and has also shown activity in NRAS mutant melanoma in combination with 6-thio-2′-deoxyguanosine [207]. Phenformin and metformin, biguanides used in the treatment of type 2 diabetes, have shown antitumor activity in vitro and in vivo. The use of phenformin has shown not only to enhance the therapeutic effect of BRAF inhibitors [208] but also to inhibit MDSC activity and increase the efficacy of anti-PD1 in melanoma [209]. Benserazide, an inhibitor of the M2 splice isoform of pyruvate kinase (PK2M), which is a key enzyme for generating pyruvate and ATP in the glycolytic pathway, leads to the inhibition of aerobic glycolysis and concurrent upregulation of OXPHOS. Since PK2M activity and aerobic glycolysis are upregulated in BRAF inhibitors resistant melanoma cells, treatment with benserazide results in a heightened sensitivity to suppressed PK2M expression both in vitro and in vivo, with potential therapeutic applications [210]. Recently, the glutamine pathway also emerged as a possible metabolic reprogramming strategy in melanoma resistant to targeted therapies [211]. In fact, BRAF-inhibitors-resistant melanoma cells increase uptake of glutamine and show overexpression of glutaminase (GLS). The mechanisms that drive this switch from glucose utilization to glutamine remain unclear, but treatment with GLS inhibitors re-sensitizes resistant cells to BRAF inhibitors [211] and also to the chemotherapeutic drug temozolomide (TMZ) [212]. Consistently, high-OXPHOS melanoma could be supported by glutamine and fatty acid oxidation via PGC-1α axis, as demonstrated also in other cancer types [213]. Preclinical evidence has shown that the inhibition of mTORC1/2 can decrease PGC-1α expression and inhibit OXPHOS. Moreover, resistance to MAPK inhibitors can be overcome by mTORC1/2 through the nuclear exclusion of MITF, and this combination has a synergistic effect only in the OXPHOS-high phenotype of melanomas [205].

Metabolic reprogramming represents an intriguing target also in uveal melanoma. Unlike its cutaneous counterpart, uveal melanoma has not gained any therapeutic benefit over the last years, and the median survival for patients with metastatic disease is less than 12 months [214]. More than 90% of uveal melanomas show monosomy of chromosome 3, a genetic feature typically associated with metastasis and poor prognosis [215]. Molecular studies have shown that elevated levels of non-mutant succinate dehydrogenase A (SDHA), a fundamental link between the TCA and OXPHOS, are the core of a distinct metabolic program that finally leads to increased biologic aggressiveness and resistance to OXPHOS inhibition [216]. Uveal melanomas frequently show guanine nucleotide-binding protein G(q) *GNAQ* and *GNA11* mutations, which enhance MEK-ERK1/2 signaling. Adaptive upregulation of OXPHOS has been described as a mechanism of resistance during treatment with MEK inhibitors in combination with CDK4/6 inhibitors. The addition of the OXPHOS inhibitor, IACS-010759, to CDK4/6 plus MEK inhibitors decreased cell growth and enhanced apoptosis, suggesting that direct OXPHOS inhibition could be an approach to optimize targeted therapy treatment in uveal melanoma [217]. In this setting, targeting the metabolic substrates and OXPHOS is an appealing therapeutic strategy since targeted therapies and immunotherapy have led to disappointing results in uveal melanoma.

## 6. Conclusions

Melanoma is a metabolic heterogeneous disease with the ability to adapt its metabolism in order to utilize a variety of fuels for energy production, facilitating tumor progression and metastasis. The significance of metabolic rewiring in melanoma is supported by growing evidence of the impact, in terms of increased efficacy, of therapeutic strategies targeting metabolic molecules in combination with standard therapies. Increasing data support the notion that metabolic phenotypes of melanoma cells depend on the contribution from both tumor intrinsic factors and extrinsic factors deriving from the TME conditions. The complex interplay between tumor metabolism and the immune system emerges as the most important aspect, as highlighted also by the numerous ongoing clinical trials specifically focused on the combination of agents targeting metabolic pathways altered in melanoma and immunotherapy (Table 1). Overall, available evidence suggests that targeting metabolic rewiring mechanisms and the metabolic crosstalk within TME represents one of the most promising and novel therapeutic strategies to overcome drug resistance or to increase therapeutic efficacy of standard treatments in specific subsets of melanoma. Prospective trials with a strong background derived from translational studies exploring both circulating and tissue metabolic biomarkers will lead to the discovery of better and more effective therapeutic combinations for the treatment of metastatic melanoma patients.

## Figures and Tables

**Table 1:** Overview of the main active and recently concluded clinical trials of drugs targeting tumor metabolism in melanoma (source: clinicaltrials.gov; accessed on 17 April 2021).

Trial Name, NCT Number	Phase	Condition(s)	Drug(s)	Metabolic Target(s)	Objective(s)	Status
NCT03207867	II	Advanced solid tumors DLBCL	NIR178 Spartalizumab (PDR001)	ADO	ORR, DCR, DOR PFS AEs PK Changes in the immune infiltrate Presence of PDR001 Ab	Active, recruiting
NCT03047928	I/II	Advanced melanoma	PD-L1/IDO peptide vaccine Nivolumab	PD-L1-IDO	AEs Treatment-related immune responses ORR OS, PFS	Active, recruiting
NCT04007588	II	Resectable stage III/IV melanoma	Linrodostat (BMS986205) Nivolumab Ipilimumab	IDO	mPCR RFS, OS Changes in the immune infiltrate AEs	Withdrawn (slow accrual)
NCT02073123	I/II	Advanced melanoma	Indoximod Nivolumab Pembrolizumab Ipilimumab	IDO	AEs ORR, DCR Mechanisms of activity/resistance to IDO/CTLA-4 inhibitor therapy OS, PFS	Completed
ECHO-208, NCT03347123	I/II	Advanced solid tumors	Epacadostat Nivolumab Ipilimumab Lirilumab	IDO KIR2DL1/2L3	AEs ORR, DOR PFS	Completed
NCT04148937	I	Advanced solid tumors	LY3475070 Pembrolizumab	CD73	DLT PK ORR, DOR PFS	Active, recruiting
PANAMA, NCT02702492	I	Advanced solid tumors NHL	KPT-9274 Niacin ER Nivolumab	PAK4 NAMPT	MTD	Active, recruiting

Abbreviations: Ab, antibodies; ADO, adenosine; AEs, adverse events; CTLA-d1, cytotoxic T lymphocyte Antigen 4; DCR, disease control rate; DLBCL, diffuse large B cell lymphoma; DLT, dose-limiting toxicity; DOR, duration of response; ER, extended release; IDO, indoleamine 2,3-dioxygenase; KIR2DL1/2L3, killer-cell immunoglobulin like receptor; mPCR, major pathologic response; MTD, maximum tolerated dose; NAMPT, nicotinamide phopshorybosiltransferase; NHL, non-Hodgkin lymphoma; ORR, objective response rate; OS, overall survival; PD-L1, programmed cell death ligand 1; PK, pharmacokinetics; PFS, progression-free survival; RFS, relapse-free survival.
